# Residual Efficacy of a Deltamethrin Emulsifiable Concentrate Formulation against *Rhyzopertha dominica* (F.) and *Sitotroga cerealella* (Oliver) after Partial Treatment of Brown Rice

**DOI:** 10.3390/insects10040095

**Published:** 2019-04-01

**Authors:** Frank H. Arthur

**Affiliations:** USDA, ARS, Center for Grain and Animal Health Research, 1515 College Avenue, Manhattan, KS 566502, USA; frank.arthur@ars.usda.gov

**Keywords:** stored rice, insects, insecticides, management

## Abstract

*Rhyzopertha dominica* (Fab.), the lesser grain borer, and *Sitotroga cerealella* (Olivier), the Angoumois grain moth, are internally feeding stored product insects that can infest raw grains. In this test, brown rice was treated with 0.5 and 1.0 ppm of a new emulsifiable concentrate (EC) formulation of the pyrethroid deltamethrin and stored for 12 months. One day after treatment, and every 3 months for 12 months, treated rice was mixed with untreated brown rice in the following ratios: 0:100 (untreated controls), 10:90, 25:75, 50:50, 75:25, and 100:0 (all treated). Bioassays were conducted by exposing 10 parental adults of each species on the rice mixtures and assessing progeny production, feeding damage, and weight loss. The progeny of *S. cerealella* ranged from 105.6 F_1_ adults on untreated brown rice to 69.4 F_1_ adults on 100% treated rice, but there was little feeding damage or weight loss. The progeny production of *R. dominica* declined from 177.4 F_1_ adults on untreated rice to 9.8 F_1_ adults on 100% treated rice. Weight loss and feeding damage were correlated with progeny production. The results show that the new deltamethrin formulation could be used for protection of brown rice, but *S. cerealella* may be less susceptible to deltamethrin compared to *R. dominica*.

## 1. Introduction

Rice is cultivated in the south-central United States (USA), principally in Arkansas, southwestern Louisiana, eastern Texas, extreme southeast Missouri, parts of Mississippi on the eastern side of the Mississippi river, as well as in the northern part of the central valley of California [1]. It can be stored as rough (paddy) rice in bins or elevators or as milled rice (either white or brown) in bulk storage or in bags. The rough rice husk offers some degree of protection from insect damage, and often a crack or a split in the husk will help facilitate the entry of stored product insects for feeding or oviposition [2,3,4]. 

In the USA, several insecticides are registered for direct application to rough rice and brown rice. These include Storicide II^®^, which is a mixture of 3 ppm of the organophosphate chlorpyrifos-methyl and 0.5 ppm of the pyrethroid deltamethrin, Centynal^®^, which contains 0.5 ppm of deltamethrin, Diacon IGR+^®^, which is a mixture of the insect growth regulator (IGR) methoprene and either 0.5 or 1.0 ppm of deltamethrin, and Diacon IGR^®^, which contains only methoprene. Primary insect pests of rough rice and brown rice include *Rhyzopertha dominica* (Fab.), the lesser grain borer, *Sitophilus oryzae* (L.), the rice weevil, and *Sitotroga cerealella* (Olivier), the Angoumois grain moth. The female *S. oryzae* lays an egg directly inside the kernel, and thus the larvae are protected from exposure to residues from an insecticide treatment. In contrast, females of *R. dominica* or *S. cerealella* lay eggs on the exterior of a grain kernel, and the larva hatches and bores into the kernel, where it completes development to the adult stage. There is a brief time window where a larva will be exposed to an insecticide [4,5], whether it is a contact toxicant or an IGR.

In previous studies with the IGR methoprene, the progeny production of *R. dominica* was completely suppressed for periods up to two years on either rough rice or brown rice [5]. However, some larvae of *S. cerealella* were able to survive the exposure to the methoprene residues and emerge as adults [5]. It may be necessary to combine another insecticide with methoprene to obtain complete control of *S. cerealella*. Combination treatments of various insecticides have been shown to increase the efficacy of insecticides with different modes of action when applied for control of stored product insects [6,7,8,9].

Grain protectants are applied to grains as they are loaded into storage or, in the case of brown rice, when they are being loaded into bags. The insecticides are applied at a specified volume rate, for example, according to the label for Diacon IGR+^®^: in this case, either 212 or 424 mL of the formulation (depending on the desired label rate) in 18.9 L of water is used to cover 20.45 metric tons (MT) of rice. With this low water volume spray rate, it is likely that there is differential deposition when rice is treated. In actual storage practices, it is possible that treated rice can be mixed in various proportions with untreated rice. The objectives of this study were to: 1) compare the responses of *R. dominica* and *S. cerealella* larvae exposed on brown rice, with different proportions of brown rice treated with 0.5 or 1.0 ppm of a new deltamethrin emulsifiable concentrate (EC) formulation, and 2) determine the residual efficacy after rice was stored for different time periods. The test was conducted using brown rice, because brown rice is more susceptible to insect damage compared to rough rice [6].

## 2. Materials and Methods

### 2.1. General Information

This test was conducted at the USDA-ARS-Center for Grain and Animal Health Research (CGAHR), Manhattan, KS, USA. The *R. dominica* and *S. cerealella* used in the test were from pesticide-susceptible laboratory strains that had been maintained at the CGAHR for more than 30 years. However, both species had been cultured on brown rice for only about four years. Both strains were reared on whole-kernel mixed-variety long-grain brown rice that was originally obtained from a commercial rice mill. All cultures were reared in total darkness inside an incubator set at 27 °C, 60% R.H.

### 2.2. Insecticide and Treatment Procedures

The insecticide was a new EC formulation of the pyrethroid deltamethrin (Centynal^®^), obtained from Central Life Sciences (Schaumberg, IL, USA). The formulation contained 50 mg of active ingredient [Al]/mL. Two concentrations of the insecticide were used in the test, 0.5 and 1.0 ppm. Label specifications for the commercial formulation at the time the experiment was initiated included only the 0.5 ppm rate, which corresponded to 202 mL of the formulation in 18.9 L to cover 20,454 kg of rice. The equivalent volume to treat 1 kg of rice was 0.9 mL. In this test, the amount of rice that comprised a replicate was 2.7 kg, which required 2.4 mL of formulated spray. For each of five replicates at both rates, the 2.7 kg sample was spread out on a 0.6 × 0.3 m piece of cardboard inside a fume hood. The equivalent label ppm rate was prepared by mixing 0.35 mL of the deltamethrin EC in 25 mL water and then using a Badger 100 artist’s air brush (Badger Corporation, Franklin Park, IL, USA) to mist 2.4 mL of the solution onto the rice. About one-third of this amount was sprayed onto the rice, then the rice was hand-mixed, and the process repeated until the entire amount was dispensed. Separate solutions were formulated for each replicate. For the 1.0 ppm rate, the amount of deltamethrin EC formulation was doubled to 0.7 mL, which was mixed with 25 mL water, and the rice prepared and treated as described above. The 1.0 ppm rate of deltamethrin was used to gather data to support a potential change to the insecticide label to allow use this higher rate. After each replicate was treated, it was placed in an 18.9 L plastic bucket and transferred to the floor of an empty metal grain bin at the CGAHR. This entire process was initiated and completed on 9 March 2016.

### 2.3. Bioassay Procedures

One day after treatment, the buckets containing the treated rice were removed from the grain bin and brought to a laboratory at the CGAHR. The desired mixture treatments were prepared using the following six percentage ratios of treated and untreated rice: 0:100 (untreated controls), 10:90, 25:75, 50:50, 75:25, and 100:0 (all rice treated). The individual exposure arena was a 120 mL plastic vial. 

Each replicate of each concentration required two sets of six vials. Treated and untreated rice was added to the vials, and each vial was hand-shaken after the rice was added to mix the treated and untreated rice. On one set of six vials, 10 mixed-sex one-week-old *R. dominica* adults were added to each vial, while 10 newly emerged *S. cerealella* adults were added to the second set of six vials. The vials containing the two species were then transferred to an environmental chamber set at 27 °C and 60% R.H. The buckets containing the remaining treated rice were then returned to the grain bin.

The vials containing *R. dominica* and *S. cerealella* were held for 8 and 5 weeks, respectively, then frozen for several days at −18 °C to kill all adults. Afterwards, the vials were removed from the freezer and allowed to warm in the laboratory. The contents of each vial were weighed by emptying the entire contents of a sample vial into a clean vial, then the adults and feeding damage were removed. The feeding damage was the insect frass and ground rice that was produced through feeding by original parental adults and larvae of *R. dominica* and developing larvae of *S. cerealella*. The rice that was left after the insects and the feeding damage were removed was weighed a second time to determine sample weight loss. The feeding damage was also weighed again separately from the number of F1 progeny. The number of offspring was tabulated at the end of this process. The buckets containing rice were stored in grain bins for a total of 12 months, and the entire sampling process described above was repeated every 3 months.

### 2.4. Statistical Analysis

Data were analyzed by species, deltamethrin rate, mixture percentages, and month as main effects, with variables of interest being percentage sample weight loss, number of F1 progeny (total number of adults minus the original 10 parental adults), and weight of feeding damage. Mixed-model procedures under the Statistical Analysis System (Version 9.2, SAS Institute, Cary, NC, USA) were used to determine the significance of the main effects and all associated interactions. One-way ANOVA procedures were used to determine the significance within levels of the main effects. Curves were fit to the data using Table Curve software (Table Curve 2D version 5.1, Systat Software, San Jose, CA, USA).

## 3. Results

An initial analysis showed that species, deltamethrin rate, mixture percentage, and month (hereafter termed months) significantly affected progeny production, with *p* < 0.001 (F = 15.1, df = 1587; F = 10.9, df = 1,587; F = 45.0, df = 5587; F = 5.3, df = 4587, respectively). Data were then sorted by species, and the main effects of deltamethrin rate, mixture percentage, and months were analyzed. 

**Figure 1:**
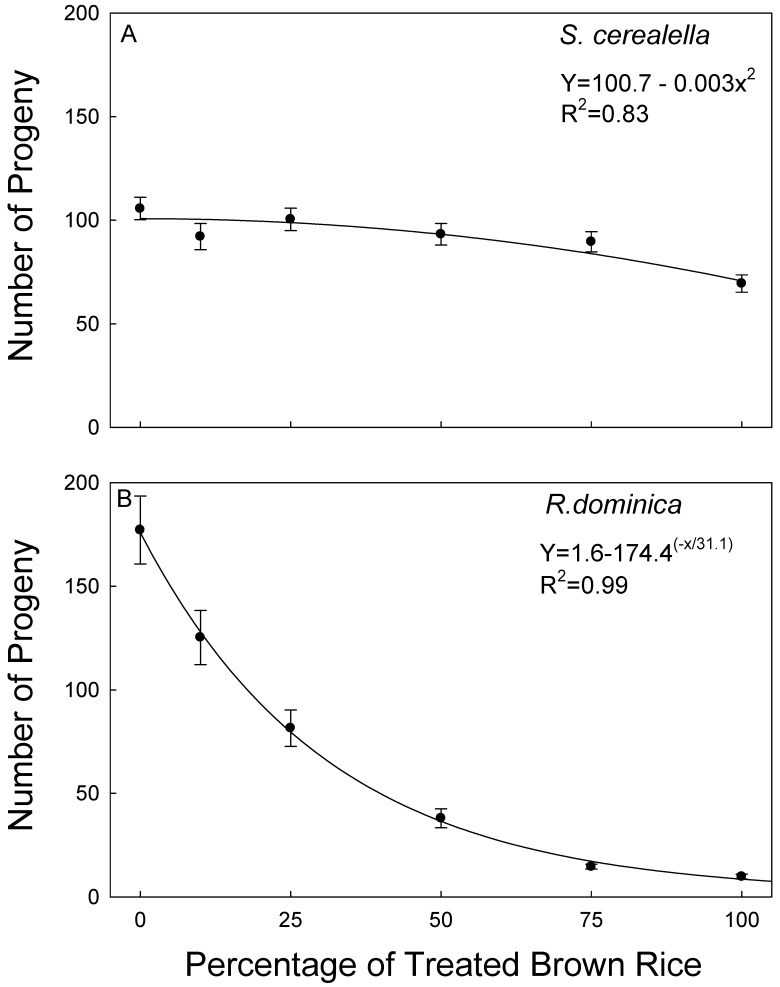
Equations and R^2^ values for number of progenies of *Sitotroga cerealella* (**A**) and *Rhyzopertha dominica* (**B**) produced from exposure of 10 mixed-sex parental adults on ca. 80 g of mixtures of untreated and treated brown rice. The percentage of treated brown rice ranged from 0 (untreated controls) to 100%. Data averaged over 15 months.

The effect of mixture percentage (the various combinations of treated and untreated rice) was significant at *p* < 0.001 for *S. cerealella* (F = 6.2, df = 1288), but neither deltamethrin rate nor months was significant (F = 0.8, df = 5,288, *p* = 0.21, F = 1.2, df = 4,288, *p* = 0.31). Even though the overall ANOVA was significant for deltamethrin rate, there was no significance with respect to progeny production of *S. cerealella* at any of the mixtures of treated and untreated brown rice. Progeny production averaged 105.6 ± 5.4 (SEM) on untreated brown rice, and there was a generalized linear decrease in progeny with increasing percentage of treated brown rice in the bioassays (Figure 1A). However, progeny production averaged 69.4 ± 4.2 when all the rice was treated. Thus, because of the lack of significance for the main effects of deltamethrin rate and months on progeny production, data were then combined with those related to months and insecticidal rate for analyses of sample weight loss and feeding damage. There was no significance with respect to mixture percentage for weight loss (F = 1.2, df = 1,93, *p* = 0.312); therefore, data were averaged over all samples. No feeding damage was recorded for *S. cerealella* during the study, so no further analysis was conducted. 

**Figure 2:**
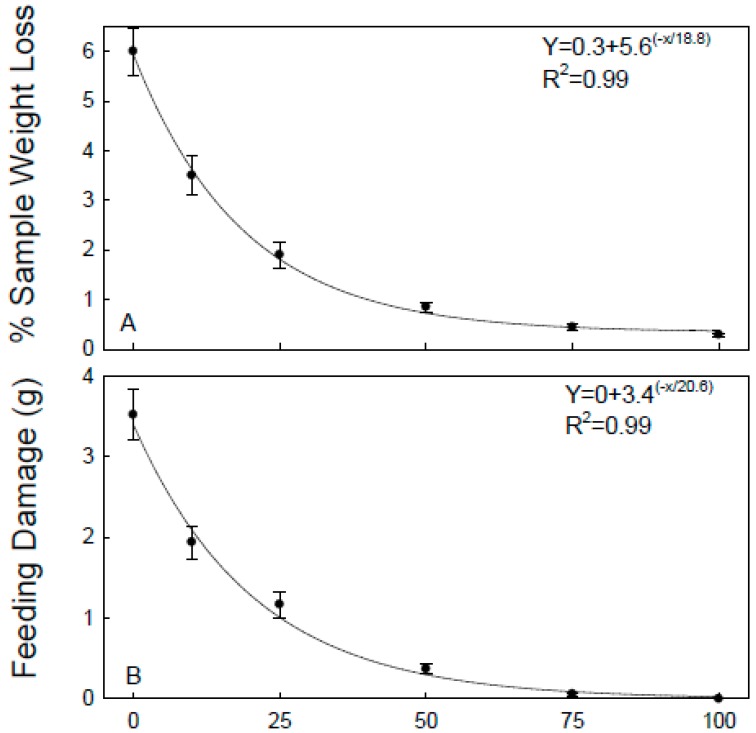
Equations and R^2^ values percentage of sample weight loss (**A**) and amount of feeding damage in grams (**B**) caused by infestations of *R. dominica* on mixtures of untreated and treated brown rice. Data averaged over 15 months.

**Figure 3:**
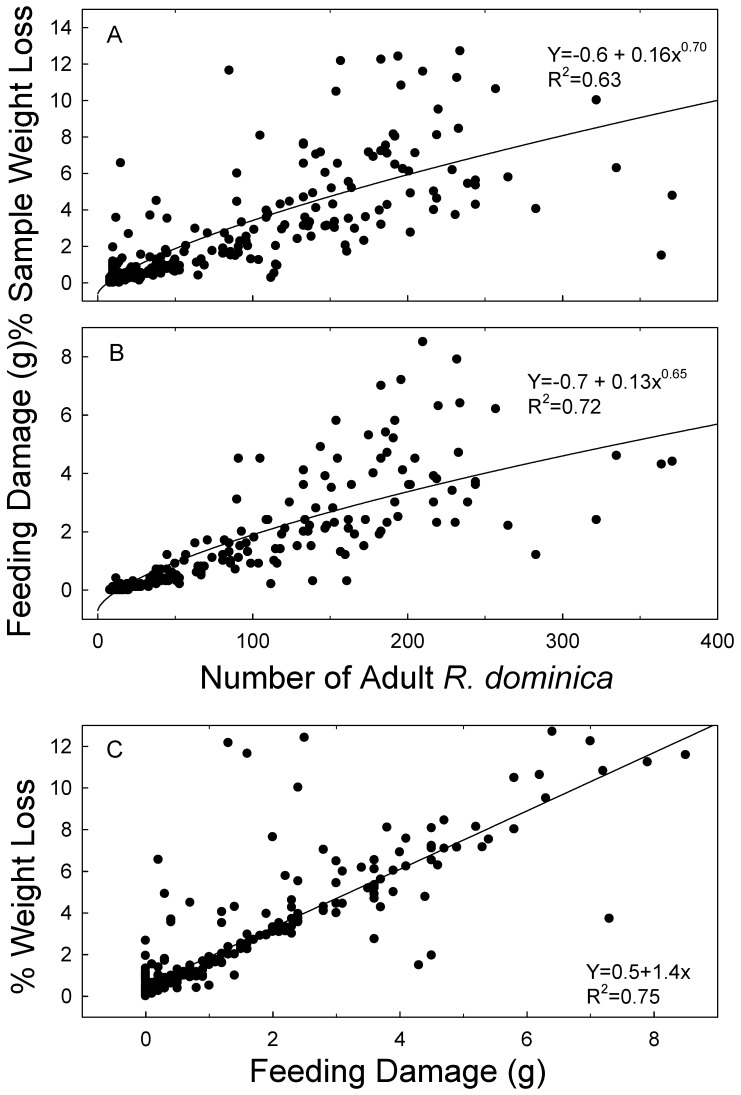
Equations and R^2^ values for percentage of sample weight loss (**A**) and feeding damage (**B**) with respect to adult progeny of *R. dominica*, and equation and R^2^ values for sample weight loss (y axis) and feeding damage (x axis) (**C**). The data plotted are all data collected for *R. dominica* over 15 months.

The main effects of deltamethrin rate, mixture percentage, and months were all significant for *R. dominica* (F = 13.2, df = 1288, *p* = 0.030; F = 59.0, df = 5,288, *p* < 0.001; F = 6.8, df = 4288, *p* < 0.001, respectively). However, for the main effect of months, when regressions were run with months as the dependent variable for each mixture percentage, there was only one instance in which the effect was significant at *p* < 0.05. Thus, the significant differences between monthss for *R. dominica* were due to random variation and not related to a decline of insecticidal efficacy over time. Data related to monthss were then combined for analysis. There was only one instance of significance for *R. dominica* (*p* ≥ 0.05 in all but one case). Data were therefore combined again considering treatment and re-analyzed. There was a non-linear decrease of *R. dominica* progeny as the percentage of treated brown rice increased, from a high number of offspring (177.4 ± 16.4) on untreated brown rice to a low number (9.8 ± 1.2) when all the rice was treated (Figure 1B). The effect of mixture percentage was significant at *p* < 0.001 for both weight loss and feeding damage in *R. dominica* (F = 63.3, df = 5292; F = 65.3, df = 5292; respectively), and, for both, there was a corresponding non-linear increase with increasing percentage of treated rice (Figure 2A,B). Sample weight loss and feeding damage were both strongly positively correlated with the number of F1 progeny (Figure 3A,B). Sample weight loss was also positively correlated with feeding damage (Figure 3C). 

## 4. Discussion

The results of this study show that, while the new deltamethrin formulation will control *R. dominica* and reduce progeny production in *S. cerealella*, a partial treatment of a grain mass may not give optimum control. The presence of the untreated brown rice in the mixture offered a means of escape for the exposed parental adults and the emerged F1 progeny. In a previous test with the deltamethrin SC formulation, Kavallieratos et al. [10] exposed adults of six beetle species, including *R. dominica*, on brown rice treated at the rate of 0.5 ppm and mixed with various percentages of untreated brown rice. The mortality of parental *R. dominica* adults did not exceed 5%, which was most likely related to the short exposure times, but there were significant decreases in progeny production of *R. dominica* as the percentage of treated rice increased in the mixtures. The effects on progeny production were most likely due to the exposure of neonate larvae to insecticide residues. The larvae were affected by exposure to insecticide, so that they either were unable to penetrate the rice kernel or did not develop after they penetrated the kernel. 

However, there was about a 35% reduction of *S. cerealella* progeny production even in the 100% treated brown rice, as compared to progeny in untreated controls, in contrast to the near complete reduction observed for *R. dominica*. This differential susceptibility of *S. cerealella* compared to *R. dominica* was also noted by Arthur [5] in a study with the IGR methoprene on both rough rice and brown rice, which utilized the same laboratory strains. Either the *S. cerealella* strain used in the study was more tolerant to insecticides compared to the *R. dominica* strain, or the larvae of *S. cerealella* take less time to enter the rice kernel compared to those of *R. dominica*, thus resulting in less exposure to the insecticide residues. It is also possible that *R. dominica* oviposition was more affected by the exposure to deltamethrin residues compared to *S. cerealella* oviposition. The current study also noted little feeding damage caused by *S. cerealella*, with no correlation between progeny numbers, feeding damage, and weight loss. This lack of feeding damage occurred even with obvious holes created by the adults when they emerged from the kernels.

Several factors may cause stored grain to contain a mixture of treated and untreated seeds, such as inadequate coverage while treating grain during the loading process and mixing of treated and untreated grain when grains from different locations are mixed. This can occur when grains are transferred from one location to another, such as between on-farm storage and a grain elevator. 

Another way treated and untreated grains can be mixed is through the practice of “top dressing”, which corresponds to treating only the surface portion of a grain mass. Laboratory and field tests produce mixed results, in that both increases in mortality or reductions of progeny were reported for surface treatments involving top dressing compared to controls, because top dressing offers insects ample opportunities to penetrate through the treated surface into the untreated portion of the grain [11,12]. Furthermore, as discussed in a study with diatomaceous earth [13], stored product insects that penetrate through a treated surface in a grain mass may be able to oviposit in the untreated layers before they die. 

The new deltamethrin EC formulation is a promising insecticide that should be considered for inclusion into pest management programs for stored product insects infesting brown rice in the US. Brown rice can be marketed directly to consumers and is now classified as a whole grain by the US Food and Drug Administration (FDA). However, testing is warranted for additional pest species, such as *Sitophilus* spp., that are common pests of stored products. Also, *Tribolium castaneum* (Herbst), the red flour beetle, can complete development on brown rice at temperatures ranging from 20 to 35 °C [14]. This insect pest is cosmopolitan in distribution, has a wide range of food habits, and is a frequent pest of rice mills [15,16,17,18]. 

## 5. Conclusions

The results of this study show that the new deltamethrin EC formulation could be used to protect stored rough rice from damage caused by Rhyzopertha dominica or Sitotroga cerealella. The level of protection would appear to be at least for one year. However, *S. cerealella* is less susceptible to insecticides compared to *R. dominica*, therefore adjustments to treatment protocols may be necessary for this species.
